# Effect of Dimethylaminohexadecyl Methacrylate With and Without Nystatin on the Hardness, Water Sorption, and Water Solubility of Polymethyl Methacrylate (PMMA) Denture Base Resin

**DOI:** 10.7759/cureus.96779

**Published:** 2025-11-13

**Authors:** Tahir Ali Khan, Mamoona Shah, Nighat Shafiq

**Affiliations:** 1 Dental Materials, Sardar Begum Dental College and Hospital, Gandhara University, Peshawar, PAK; 2 Dental Materials, Bacha Khan Dental College, Mardan, PAK; 3 Oral Biology, Khyber College of Dentistry, Peshawar, PAK

**Keywords:** dmahdm, hardness, nystatin, pmma, solubility, water sorption

## Abstract

Purpose: Polymethyl methacrylate (PMMA) denture base resins provide a surface conducive to microbial colonization and biofilm formation, which can precipitate denture-related stomatitis. Incorporation of antimicrobial agents, such as dimethyl amino-ethyl hexadecyl dimethacrylate (DMAHDM) and nystatin, reduces this risk; however, their inclusion could alter the physicomechanical properties of PMMA. This study aimed to evaluate the effects of incorporating DMAHDM, alone or with nystatin, into PMMA denture base resin on Vickers microhardness, water sorption, and solubility.

Methodology: Heat-polymerized PMMA specimens were prepared as control (A) and experimental groups with 5%, 10%, or 20% DMAHDM (B1-B3) with/without 500,000 IU nystatin (C1-C3). Hardness (n = 10/group) was tested using a calibrated Vickers indenter (300 g, 10 seconds). Water sorption (n = 5) and solubility (n = 5) were measured at 1 and 30 days and are reported in micrograms per square millimeter according to ISO 20795‑1. Between‑group comparisons used one‑way analysis of variance with Tukey's post hoc test (p < 0.05).

Results: All DMAHDM‑containing groups exhibited higher hardness than the control (p < 0.001); 5% DMAHDM (B1) was significantly higher than other experimental groups (p < 0.001). Sorption and solubility increased with the concentration of DMAHDM and with the addition of nystatin. Converted per‑area values remained within ISO 20795‑1 limits across groups.

Conclusion: Low levels of DMAHDM significantly improve hardness, but higher concentrations and the addition of nystatin increase water uptake and solubility, which may negatively impact long-term stability. Optimization of DMAHDM loading is warranted.

## Introduction

Edentulism, characterized by loss of all natural teeth (complete) or some teeth (partial), leads to the use of a dental prosthesis to restore oral function and aesthetics. For this purpose, an implant-retained prosthesis is the ideal solution; however, its application is restricted due to cost [1]. The alternate material of choice is polymethyl methacrylate (PMMA), which has been widely used by prosthodontics since the turn of the 20th century. PMMA possesses various favorable attributes, making it the most appropriate material for the fabrication of prostheses [2]. Despite these advantages, PMMA resin has some significant limitations. Due to its dynamic nature and exposure to numerous solvents that can be absorbed into the material, the oral environment creates particular problems for denture base materials. Fluid uptake and monomer release substantially impact the physical and mechanical properties of restorative materials, including flexural and impact strength, poor surface hardness, and insufficient antimicrobial activity [3-5]. Therefore, water sorption and solubility are the crucial problems that compromise the longevity of the denture base [6].

Water sorption refers to the capacity of the material to absorb and retain water when exposed to oral fluids due to the polarity of resin molecules. The absorbed water diffuses between the materials' macromolecules by separating the polymer chains, causing expansion. The rate of water sorption into a polymeric structure is determined by the degree of conversion, hydrophilicity, and cross-link density of the polymer network [7,8]. Water sorption of acrylic resin prosthesis works as a plasticiser, influencing the mechanical and physical parameters such as transverse strength, hardness, and fatigue limit. It also reduces the lifespan of a denture by producing internal stresses, which can lead to an increased potential for fractures [5,6,9].

Solubility is expressed by the proportion of material dissolved by the polymer. Denture base resins generally have minimal solubility, which occurs due to the leaching out of remnants of unreacted monomers and initiators into the oral fluids. In contrast, in denture soft lining materials, additional leaching may occur due to the presence of plasticizers. However, these monomers can occasionally cause a soft tissue reaction. Water sorption and solubility both have a negative impact on the durability of the denture. Hardness assesses a material's capacity to endure persistent indentation or penetration and predicts wear resistance. The hardness of a material indicates its resistance to scratching and ease of finishing [10]. A material with higher surface hardness is considered more wear-resistant. A significant issue is the abrasive wear of acrylic resin removable partial and complete dentures during function and mechanical cleaning. As a result, the surface hardness of the acrylic resin degrades with time, leading to increased mechanical and chemical wear of the denture base components [11].

Different researchers have studied dimethyl amino-ethyl hexadecyl dimethacrylate (DMAHDM), an antimicrobial monomer of quaternary ammonium salt (QAM). DMAHDM with a 16-carbon alkyl chain exhibits potent broad-spectrum antimicrobial activity when incorporated into denture base resin. Its positively charged QAM group interacts with the negatively charged bacterial membrane, disrupting its integrity and causing cell lysis, while the long alkyl chain enhances membrane penetration and antimicrobial efficacy. Besides directly killing bacteria, DMAHDM also inhibits biofilm formation by preventing bacterial adhesion [12-14]. The incorporation of synthesized DMAHDM (16 chain lengths) in concentrations of 5% and 10% in dental resin was evaluated for hardness before and after storage. In dry storage after 24 hours, the specimens containing 10% DMAHDM had hardness values lower than those of the control group [15].

Khan et al. evaluated the effect of nystatin and DMAHDM in combination on the antifungal and mechanical properties of acrylic denture base resin [1]. There has been no previous research on incorporating conventional PMMA with DMAHDM and nystatin for their hardness, water solubility, and water sorption. This study evaluated how incorporating DMAHDM, alone or with nystatin, influences the hardness, water sorption, and solubility of PMMA. The null hypothesis was that there would be no effect of DMAHDM alone or with nystatin on acrylic resin's hardness, solubility, and water sorption.

## Materials and methods

This in vitro experimental study was conducted at Sardar Begum Dental College and Hospital, Peshawar, Pakistan, over a period of five months. A total of seven groups were prepared, including one control (A) and six experimental groups (B1-B3 and C1-C3). For the hardness test, 10 specimens were used per group, while solubility and water sorption tests were conducted with five specimens per group. The selected sample sizes are consistent with prior in vitro studies on PMMA denture base resins and are expected to provide sufficient statistical power (≥80%) to detect differences in hardness, solubility, and water sorption between groups at α = 0.05.

Materials

Heat‑cure PMMA (MEADWAY, Bracon, UK); 2-(dimethylamino)ethyl methacrylate (DMAEMA); 1‑bromohexadecane (BHD; Sigma-Aldrich, Taufkirchen, Germany); nystatin powder (500,000 IU) (Vltavska, Roztoky, Czech Republic), and ethanol absolute (Merck KGaA, 64271 Darmstadt, Germany) were used.

DMAHDM synthesis (modified Menschutkin reaction)

The reaction was carried out between an organohalide compound and a tertiary amine group. Equimolar quantities (10 mmol each) of 2-DMAEMA and 1-BHD were mixed with 3 g of ethanol in a 20 mL glass vial. The reaction was carried out at 70°C for 24 hours with constant stirring. Once the reaction was completed, the solvent was dried under vacuum, leaving behind the precipitates of DMAHDM [14]. Spectroscopic analysis of the newly formed DMAHDM was done; proton nuclear magnetic resonance exhibited signals for aliphatic protons of the hexadecyl chain (0.85-1.4 ppm), methylene protons near ester and nitrogen groups, and a singlet at 3.0-3.3 ppm for the methyl groups on the quaternary nitrogen. Carbon-13 nuclear magnetic resonance confirmed peaks corresponding to carbonyl, aliphatic, and QAM carbons. Fourier-transform infrared spectroscopy spectra showed characteristic bands of ester carbonyl (1,720-1,740 cm⁻¹), aliphatic C-H (2,850-2,950 cm⁻¹), and C-N stretching (900-1,200 cm⁻¹), verifying QAM salt formation.

Specimen fabrication

Powder:liquid ratio 2.5:1 (w/w) for control was used. For experimental groups, DMAHDM liquid replaced an equivalent volume of monomer at 5%, 10%, or 20% (v/v). Final P:L ratios per group are as follows: A (2.5:1), B1 (2.5:1 adjusted; monomer 7.98 mL, DMAHDM 0.42 mL), B2 (monomer 7.56 mL, DMAHDM 0.84 mL), and B3 (monomer 6.72 mL, DMAHDM 1.68 mL). For nystatin groups (C1-C3), 102.99 mg of nystatin was blended with 20.98 g of PMMA by ball milling (1,200 rpm, 10 minutes) [1]. Table 1 presents the descriptions of the proportions for each group.

**Table 1:** Concentrations of DMAHDM and nystatin in various groups DMAHDM: dimethyl amino-ethyl hexadecyl dimethacrylate

Groups	Concentrations
Control group A	Conventional heat-cure acrylic resin (as supplied)
Experimental group B1	acrylic resin impregnated with 5% DMAHDM
Experimental group C1	Acrylic resin impregnated with 5% DMAHDM and nystatin 500,000 units
Experimental group B2	Acrylic resin impregnated with 10% DMAHDM
Experimental group C2	Acrylic resin impregnated with 10% DMAHDM and nystatin 500,000 units
Experimental group B3	Acrylic resin impregnated with 20% DMAHDM
Experimental group C3	Acrylic resin impregnated with 20% DMAHDM and nystatin 500,000 units

**Figure 1:**
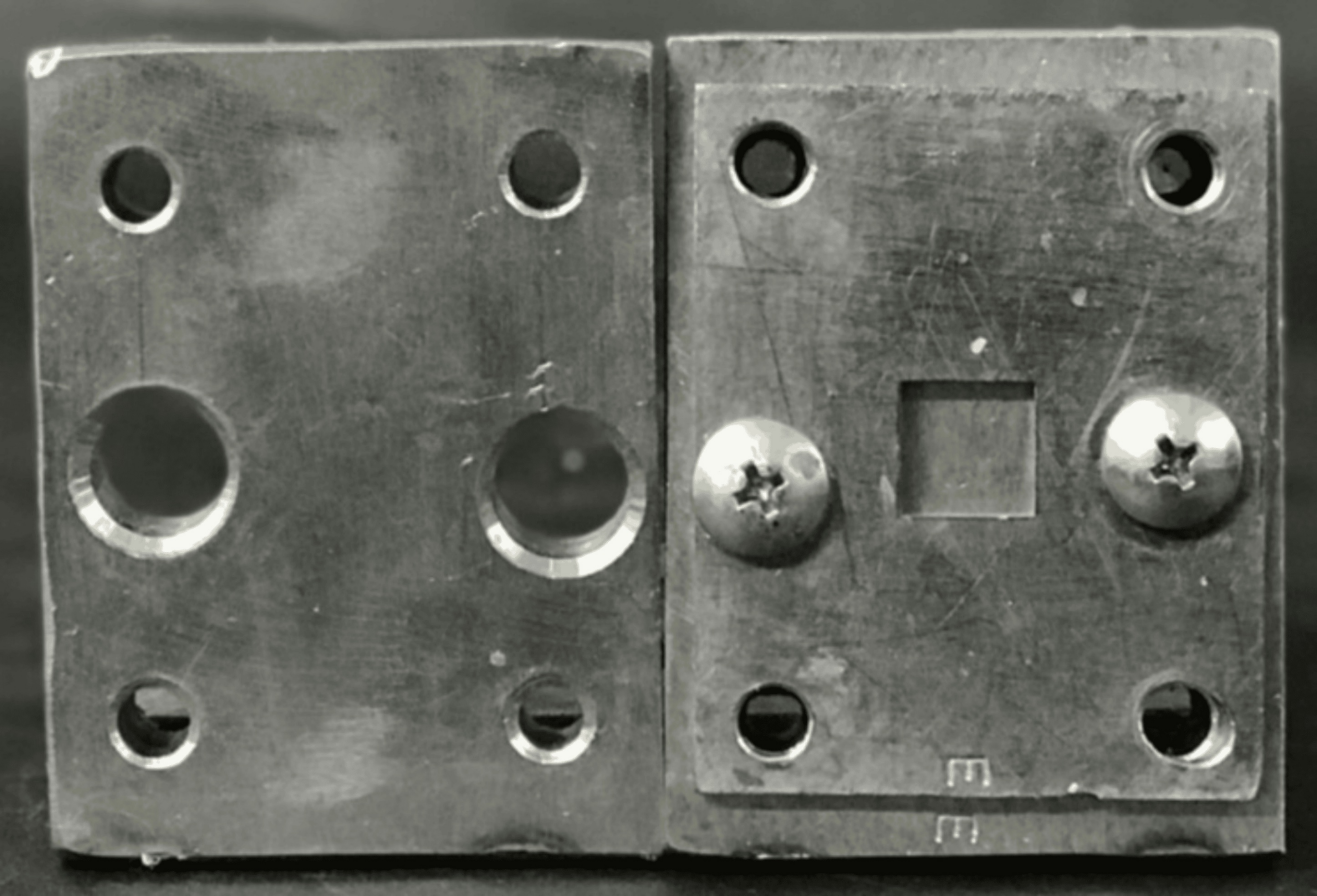
Stainless steel mold for the determination of Vickers hardness number

**Figure 2:**
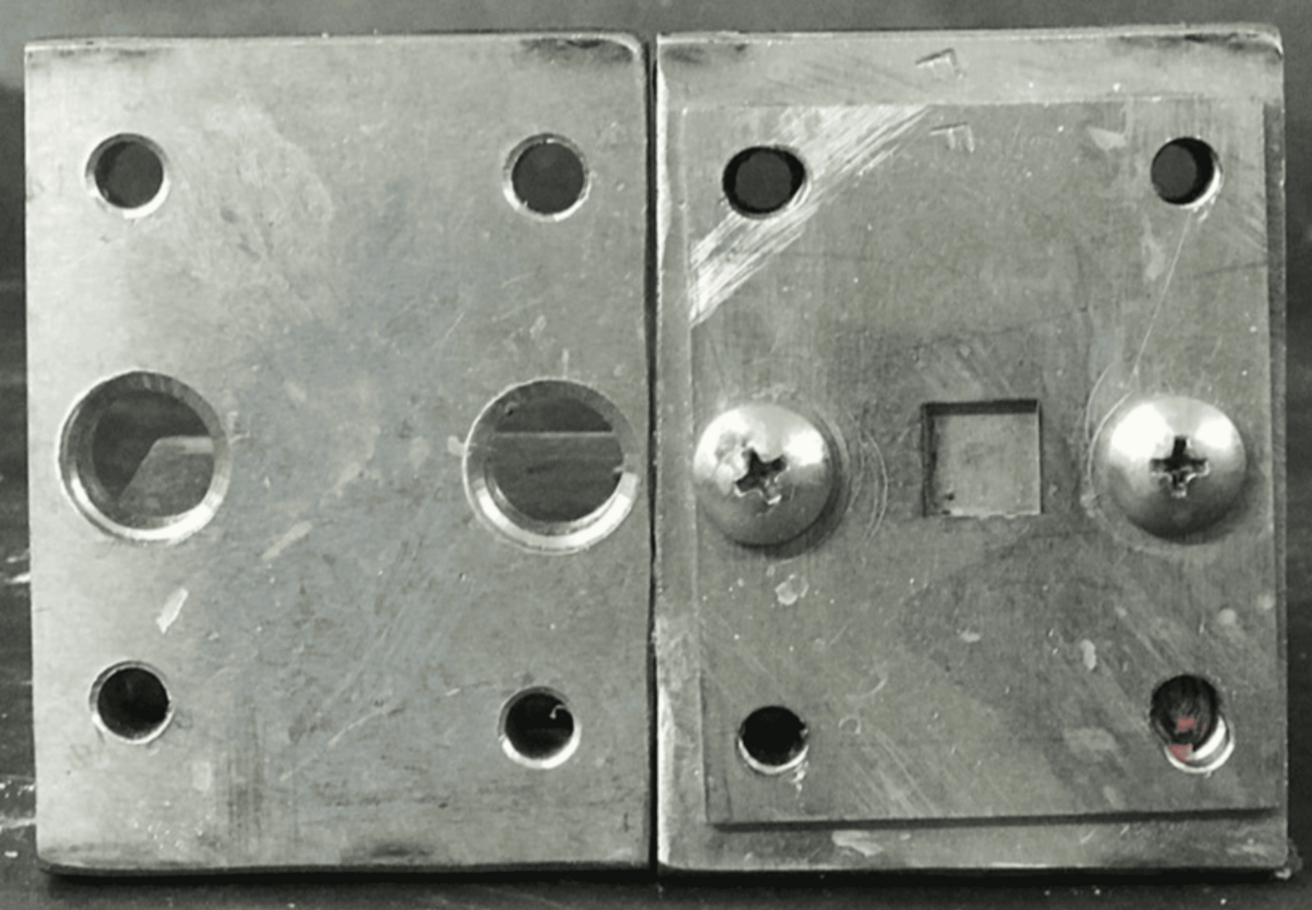
Stainless steel mold for water sorption and solubility tests

**Figure 3:**
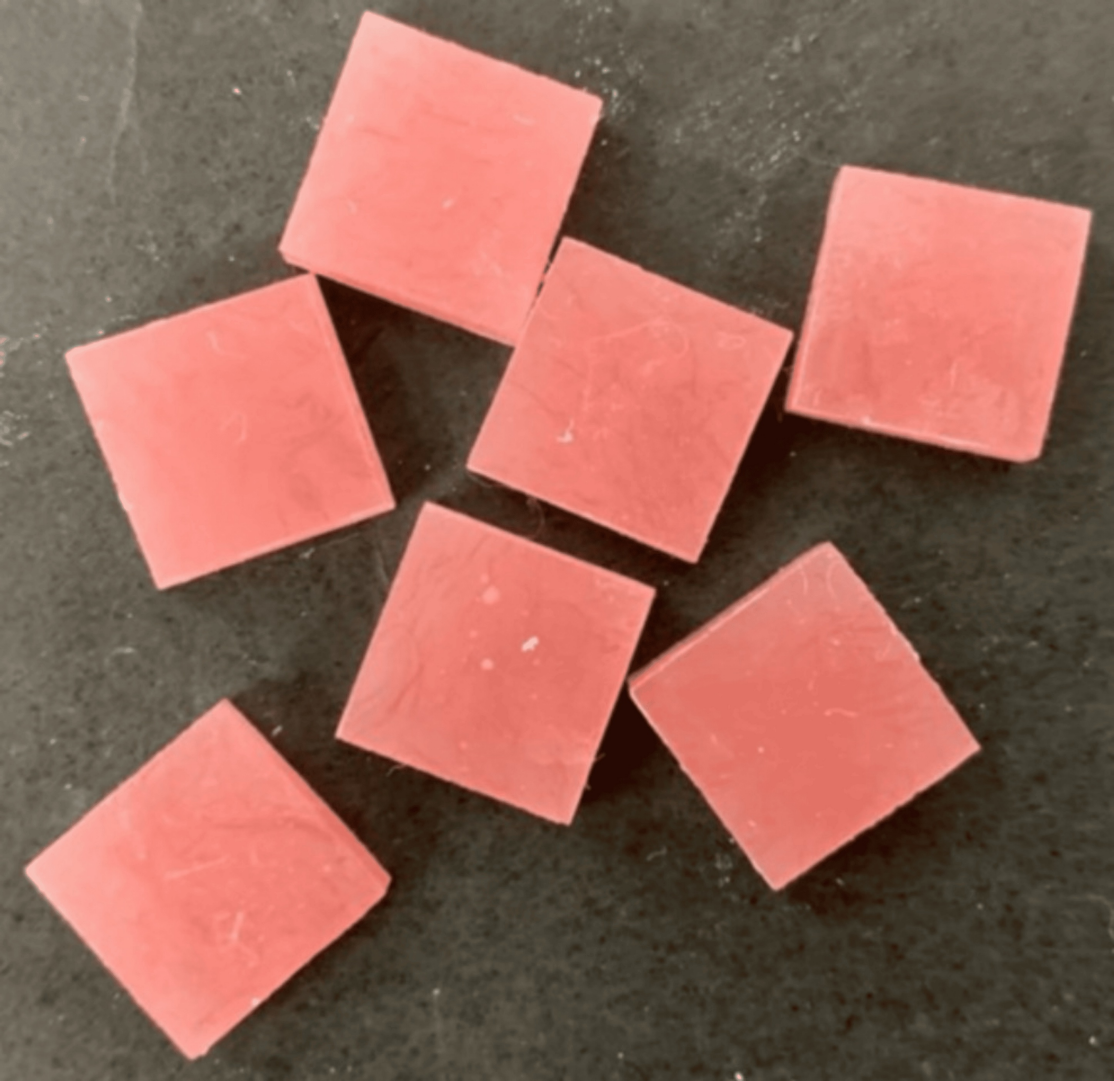
Representative image of selected specimens for Vickers hardness test

**Figure 4:**
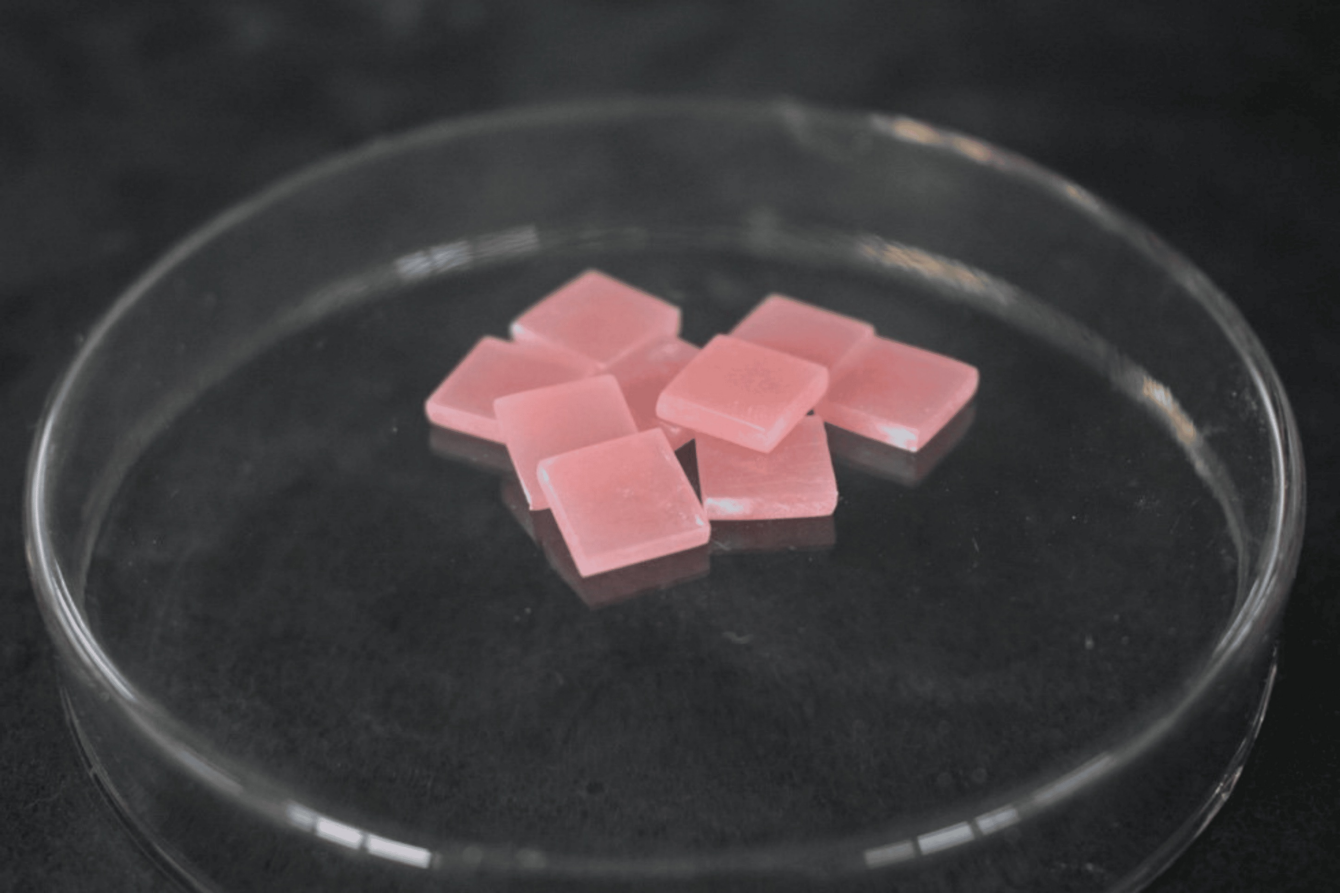
Representative image of selected specimens for water sorption and solubility tests

A stainless-steel die, prepared using a computer-aided design/computer-aided manufacturing milling machine, was used to fabricate the specimens. The dimensions of the die for the Vickers hardness test were 12 mm × 12 mm × 3 mm (Figure 1), and for testing water sorption and solubility were 10 mm × 10 mm × 2 mm (ISO 20795‑1) (Figure 2). Acrylic resin powder and monomer were mixed and packed in a flask at the dough stage. The flask was closed and subjected to a hydraulic press at 9.8 MPa for 5 minutes to remove excess material. The flask was then placed in a water-curing bath at room temperature, and the temperature was allowed to rise gradually to 100°C over 1.5 hours, which was then maintained at this temperature for 30 minutes. Once the polymerization cycle was complete, the flask was bench-cooled overnight before deflasking. The specimens were removed from the flask; excess resin was trimmed using a tungsten carbide bur and finished with 200/300/600-grit SiC and pumice polish (Figures 3, 4) [16].

Microhardness test

**Figure 5:**
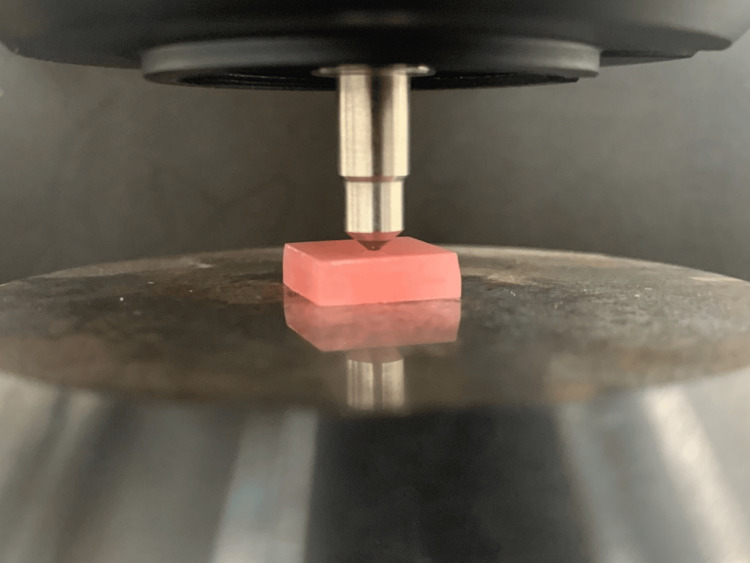
Representative image of acrylic resin sample for Vickers hardness testing

A calibrated Vickers hardness tester (Model: FH-002-0001, Serial: FH0201170213, ZwickRoell, Germany) was used for the test. A plane angle of 136° with a diamond pyramid indenter was used to perform the test. Prior to testing, the specimens were maintained at room temperature for 24 hours. To record the Vickers hardness number (VHN), a load of 300 g was applied for 10 seconds. A square base indenter was used, upon which the diamond indenter was forced onto the specimen's surface, and the diagonal length was assured optically. After applying load F, the diagonal indent lengths d1 and d2 were measured. A total of 10 indents/specimens spaced at ≥3× diagonal and ≥1 mm from edges with verified surface flatness were taken. To determine the Vickers, the mean value of each group was estimated according to ASTM E 384-22 specifications (Figure 5). Calculations of Vickers hardness were done using the equation:

$V_H = \frac{1.8544P}{d^2}$

Water sorption and solubility procedure

The prepared specimens were kept in a silica gel desiccator at 37°C to achieve a consistent mass M1 (Δm < 0.1 mg/24 hour). Dried specimens were weighed on a precision scale, and the surface area (mm^2^) of the specimen was estimated before water storage was retrieved. Ten samples per concentration were considered. All disc samples were transferred to a separate glass vessel with 20 mL of distilled water and stored in an incubator for 30 days at 37°C, where the distilled water was changed daily. After each storage period, the specimens were taken out of the water, blotted dry with absorbent paper to eliminate any apparent surface moisture, and then waved in the air for 15 seconds before being weighed again (M2). Water sorption values of each specimen were calculated in micrograms per square millimeterusing the following formula:

$W_s = \frac{M_2 - M_3}{S}$.

The specimens were again dried to a consistent mass (M3) following the same methodology as used for M1 measurement. Solubility values were calculated in micrograms per square millimeter using the following formula:

$W_{sol} = \frac{M_1 - M_3}{S}$.

Statistical analysis

The Statistical Package for the Social Sciences software, version 22 (2016; IBM Software, Armonk, NY), was utilized for data analysis. The statistical analysis involved conducting a one-way analysis of variance (ANOVA), and the comparison between groups was performed using post hoc Tukey's (honestly significant difference) tests. A significance level of p < 0.05 was deemed statistically significant.

## Results

Hardness test

One-way ANOVA indicated significant differences across groups (p < 0.001) (Table 2). Post hoc Tukey's test revealed that the VHN of all experimental groups (B1-B3, C1-C3) was higher than that of control A (p < 0.001). Among experimental groups, the hardness of group B1 (5% DMAHDM) was significantly higher than that of B2, B3, C1, C2, and C3 (all p < 0.001), while no significant difference was found among groups B2, B3, C1, C2, and C3 (Table 3).

**Table 2:** Comparison of hardness (VHN) of denture base material modified with different concentrations of DMAHDM alone and with nystatin using one-way ANOVA SD: standard deviation; ANOVA: analysis of variance; MS: mean square; SS: sum of square; df: degrees of freedom; DMAHDM: dimethyl amino-ethyl hexadecyl dimethacrylate; VHN: Vickers hardness number p values less than 0.05 are statistically significant

Groups	n	Mean ± SD	ANOVA summary						F ratio	p value
			Between groups			Within groups				
			df	MS	SS	df	MS	SS		
A	10	13.311 ± 1.16	6	20.969	125.815	63	1.343	84.580	15.619	0.000
B1	10	18.243 ± 1.35								
B2	10	15.455 ± 1.0								
B3	10	15.207 ± 1.07								
C1	10	15.277 ± 1.05								
C2	10	15.335 ± 1.02								
C3	10	15.120 ± 1.36								

**Table 3:** Post hoc multiple comparisons (Tukey’s HSD) for surface hardness of PMMA specimens across all experimental groups HSD: honestly significant difference; PMMA: polymethyl methacrylate ^*^The mean difference is significant at the 0.05 level

(I) groups	(J) groups	Mean difference (I-J)	Sig.	95% confidence interval	
				Lower bound	Upper bound
A	B1	-4.93200^*^	0.000	-6.5102	-3.3538
	B2	-2.14400^*^	0.002	-3.7222	-0.5658
	B3	-1.89600^*^	0.009	-3.4742	-0.3178
	C1	-1.96600^*^	0.006	-3.5442	-0.3878
	C2	-2.02400^*^	0.004	-3.6022	-0.4458
	C3	-1.80900^*^	0.015	-3.3872	-0.2308
B1	B2	2.78800^*^	0.000	1.2098	4.3662
	B3	3.03600^*^	0.000	1.4578	4.6142
	C1	2.96600^*^	0.000	1.3878	4.5442
	C2	2.90800^*^	0.000	1.3298	4.4862
	C3	3.12300^*^	0.000	1.5448	4.7012
B2	B3	0.24800	0.999	-1.3302	1.8262
	C1	0.17800	1.000	-1.4002	1.7562
	C2	0.12000	1.000	-1.4582	1.6982
	C3	0.33500	0.995	-1.2432	1.9132
B3	C1	-0.07000	1.000	-1.6482	1.5082
	C2	-0.12800	1.000	-1.7062	1.4502
	C3	0.08700	1.000	-1.4912	1.6652
C1	C2	-0.05800	1.000	-1.6362	1.5202
	C3	0.15700	1.000	-1.4212	1.7352
C2	C3	0.21500	1.000	-1.3632	1.7932

Water sorption testing

The water sorption data of unaged specimens suggest that control group A had the lowest mean sorption value compared to other experimental groups, while group C3 had the highest. One-way ANOVA indicated a significant difference among the groups (p < 0.001) (Table 4). Post hoc Tukey's test revealed that group A showed significantly lower solubility in comparison to groups B2, B3, C2, and C3 (all p < 0.01). This indicated that increasing the concentration of DMAHDM and nystatin also increased the water sorption value. Among DMAHDM-containing groups, B1 had a significantly lower sorption value compared to B3 (p < 0.001). For groups containing DMAHDM and nystatin, C1 was significantly lowest compared to C2 (p < 0.015) and C3 (p < 0.001) (Table 5).

**Table 4:** Comparison of water sorption of denture base material modified with different concentrations of DMAHDM alone and with nystatin using one-way ANOVA (unaged) SD: standard deviation; ANOVA: analysis of variance; MS: mean square; SS: sum of square; df: degrees of freedom; DMAHDM: dimethyl amino-ethyl hexadecyl dimethacrylate p values less than 0.05 are statistically significant

Groups	n	Mean ± SD	ANOVA summary						F ratio	p value
			Between groups			Within groups				
			df	MS	SS	df	MS	SS		
A	5	9.91 ± 0.86	6	8.306	49.833	28	0.258	7.237	32.136	0.000
B1	5	10.24 ± 0.68								
B2	5	11.29 ± 0.24								
B3	5	12.57 ± 0.54								
C1	5	10.53 ± 0.33								
C2	5	11.71 ± 0.24								
C3	5	13.44 ± 0.24								

**Table 5:** Post hoc multiple comparisons (Tukey’s HSD) for water sorption of unaged PMMA specimens across all experimental groups HSD: honestly significant difference ^*^The mean difference is significant at the 0.05 level

(I) groups	(J) groups	Mean difference (I-J)	Sig.	95% confidence interval	
				Lower bound	Upper bound
A	B1	-0.33000	0.943	-1.3499	0.6899
	B2	-1.37200^*^	0.003	-2.3919	-0.3521
	B3	-2.65800^*^	0.000	-3.6779	-1.6381
	C1	-0.61400	0.491	-1.6339	0.4059
	C2	-1.80000^*^	0.000	-2.8199	-0.7801
	C3	-3.53000^*^	0.000	-4.5499	-2.5101
B1	B2	-1.04200^*^	0.043	-2.0619	-0.0221
	B3	-2.32800^*^	0.000	-3.3479	-1.3081
	C1	-0.28400	0.972	-1.3039	0.7359
	C2	-1.47000^*^	0.002	-2.4899	-0.4501
	C3	-3.20000^*^	0.000	-4.2199	-2.1801
B2	B3	-1.28600^*^	0.007	-2.3059	-0.2661
	C1	0.75800	0.254	-0.2619	1.7779
	C2	-0.42800	0.832	-1.4479	0.5919
	C3	-2.15800^*^	0.000	-3.1779	-1.1381
B3	C1	2.04400^*^	0.000	1.0241	3.0639
	C2	0.85800	0.144	-0.1619	1.8779
	C3	-0.87200	0.132	-1.8919	0.1479
C1	C2	-1.18600^*^	0.015	-2.2059	-0.1661
	C3	-2.91600^*^	0.000	-3.9359	-1.8961
C2	C3	-1.73000^*^	0.000	-2.7499	-0.7101

For aged specimens, the trend remained the same, as one-way ANOVA indicated a significant difference among the groups (p < 0.000) (Table 6). Group A exhibited the significantly lowest sorption value among all the groups (p < 0.001). Among the DMAHDM-containing groups, B1 exhibited significantly lower sorption value than B3 (p < 0.001). For groups containing DMAHDM and nystatin, C1 exhibited the lowest sorption compared to C3 (p < 0.001) (Table 7).

**Table 6:** Comparison of water sorption of denture base material modified with different concentrations of DMAHDM alone and with nystatin using one-way ANOVA (30 days aged) SD: standard deviation; ANOVA: analysis of variance; MS: mean square; SS: sum of square; df: degrees of freedom; DMAHDM: dimethyl amino-ethyl hexadecyl dimethacrylate p values less than 0.05 are statistically significant

Groups	n	Mean ± SD	ANOVA summary						F ratio	p value
			Between groups			Within groups				
			df	MS	SS	df	MS	SS		
A	5	10.04 ± 0.54	6	8.852	53.111	28	0.161	4.518	54.864	0.000
B1	5	11.36 ± 0.39								
B2	5	11.70 ± 0.45								
B3	5	13.00 ± 0.41								
C1	5	11.47 ± 0.31								
C2	5	12.29 ± 0.35								
C3	5	14.16 ± 0.35								

**Table 7:** Post hoc multiple comparisons (Tukey’s HSD) for water sorption of 30 days aged PMMA specimens across all experimental groups HSD: honestly significant difference; PMMA: polymethyl methacrylate ^*^The mean difference is significant at the 0.05 level

(I) groups	(J) groups	Mean difference (I-J)	Sig.	95% confidence interval	
				Lower bound	Upper bound
A	B1	-1.34200^*^	0.000	-2.1478	-0.5362
	B2	-1.73800^*^	0.000	-2.5438	-0.9322
	B3	-2.98200^*^	0.000	-3.7878	-2.1762
	C1	-1.46000^*^	0.000	-2.2658	-0.6542
	C2	-2.26200^*^	0.000	-3.0678	-1.4562
	C3	-4.19000^*^	0.000	-4.9958	-3.3842
B1	B2	-0.39600	0.708	-1.2018	0.4098
	B3	-1.64000^*^	0.000	-2.4458	-0.8342
	C1	-0.11800	0.999	-0.9238	0.6878
	C2	-0.92000^*^	0.017	-1.7258	-0.1142
	C3	-2.84800^*^	0.000	-3.6538	-2.0422
B2	B3	-1.24400^*^	0.001	-2.0498	-0.4382
	C1	0.27800	0.925	-0.5278	1.0838
	C2	-0.52400	0.401	-1.3298	0.2818
	C3	-2.45200^*^	0.000	-3.2578	-1.6462
B3	C1	1.52200^*^	0.000	0.7162	2.3278
	C2	0.72000	0.103	-0.0858	1.5258
	C3	-1.20800^*^	0.001	-2.0138	-0.4022
C1	C2	-0.80200	0.052	-1.6078	0.0038
	C3	-2.73000^*^	0.000	-3.5358	-1.9242
C2	C3	-1.92800^*^	0.000	-2.7338	-1.1222

Water solubility testing

The solubility results revealed that the experimental group C3 exhibited the maximum mean value, while the control group A exhibited the minimum. One-way ANOVA exhibited a significant difference among the groups (p < 0.001) (Table 8). From the results of Tukey's post hoc analysis, it was observed that group A had significantly lower solubility than group C3 (p < 0.001), while group C3 exhibited the highest solubility values among all the groups (p < 0.05) (Table 9).

**Table 8:** Comparison of solubility of denture base material modified with different concentrations of DMAHDM alone and with nystatin using one-way ANOVA (unaged) SD: standard deviation; ANOVA: analysis of variance; MS: mean square; SS: sum of square; df: degrees of freedom; DMAHDM: dimethyl amino-ethyl hexadecyl dimethacrylate p values of less than 0.05 are statistically significant

Groups	n	Mean ± SD	ANOVA summary						F ratio	p value
			Between groups			Within groups				
			df	MS	SS	df	MS	SS		
A	5	0.67 ± 0.31	6	0.235	1.407	28	0.009	0.245	26.855	0.000
B1	5	0.72 ± 0.26								
B2	5	0.75 ± 0.04								
B3	5	0.77 ± 0.01								
C1	5	0.85 ± 0.17								
C2	5	0.89 ± 0.09								
C3	5	1.31 ± 0.13								

**Table 9:** Post hoc multiple comparisons (Tukey’s HSD) for solubility of unaged PMMA specimens across all experimental groups HSD: honestly significant difference; PMMA: polymethyl methacrylate ^*^The mean difference is significant at the 0.05 level

(I) groups	(J) groups	Mean difference (I-J)	Sig.	95% confidence interval	
				Lower bound	Upper bound
A	B1	-0.04200	0.991	-0.2295	0.1455
	B2	-0.07000	0.894	-0.2575	0.1175
	B3	-0.09800	0.648	-0.2855	0.0895
	C1	-0.17200	0.088	-0.3595	0.0155
	C2	-0.21800^*^	0.015	-0.4055	-0.0305
	C3	-0.63800^*^	0.000	-0.8255	-0.4505
B1	B2	-0.02800	0.999	-0.2155	0.1595
	B3	-0.05600	0.961	-0.2435	0.1315
	C1	-0.13000	0.327	-0.3175	0.0575
	C2	-0.17600	0.077	-0.3635	0.0115
	C3	-0.59600^*^	0.000	-0.7835	-0.4085
B2	B3	-0.02800	0.999	-0.2155	0.1595
	C1	-0.10200	0.606	-0.2895	0.0855
	C2	-0.14800	0.196	-0.3355	0.0395
	C3	-0.56800^*^	0.000	-0.7555	-0.3805
B3	C1	-0.07400	0.867	-0.2615	0.1135
	C2	-0.12000	0.419	-0.3075	0.0675
	C3	-0.54000^*^	0.000	-0.7275	-0.3525
C1	C2	-0.04600	0.985	-0.2335	0.1415
	C3	-0.46600^*^	0.000	-0.6535	-0.2785
C2	C3	-0.42000^*^	0.000	-0.6075	-0.2325

For the aged specimens, the observed trend remained consistent, with one-way ANOVA indicating a statistically significant difference among the groups (p < 0.001) (Table 10). Post hoc analysis revealed that group A exhibited significantly lower solubility than group C3 (p < 0.001), while no significant differences were noted relative to groups B1, B2, B3, and C2. Conversely, group C3 exhibited the highest solubility values among all groups (p < 0.001) (Table 11).

**Table 10:** Comparison of solubility of denture base material modified with different concentrations of DMAHDM alone and with nystatin using one-way ANOVA (30 days aged) SD: standard deviation; ANOVA: analysis of variance; MS: mean square; SS: sum of square; df: degrees of freedom; DMAHDM: dimethyl amino-ethyl hexadecyl dimethacrylate p values of less than 0.05 are statistically significant

Groups	n	Mean ± SD	ANOVA summary						F ratio	p value
			Between groups			Within groups				
			df	MS	SS	df	MS	SS		
A	5	0.68 ± 0.04	6	0.203	1.216	28	0.003	0.072	79.084	0.000
B1	5	0.71 ± 0.02								
B2	5	0.72 ± 0.04								
B3	5	0.74 ± 0.01								
C1	5	0.79 ± 0.06								
C2	5	0.86 ± 0.06								
C3	5	1.26 ± 0.08								

**Table 11:** Post hoc multiple comparisons (Tukey’s HSD) for solubility of 30 days aged PMMA specimens across all experimental groups HSD: honestly significant difference; PMMA: polymethyl methacrylate ^*^The mean difference is significant at the 0.05 level

(I) groups	(J) groups	Mean difference (I-J)	Sig.	95% confidence interval	
				Lower bound	Upper bound
A	B1	-0.03000	0.963	-0.1316	0.0716
	B2	-0.04400	0.810	-0.1456	0.0576
	B3	-0.06400	0.437	-0.1656	0.0376
	C1	-0.11400^*^	0.020	-0.2156	-0.0124
	C2	-0.19000^*^	0.000	-0.2916	-0.0884
	C3	-0.58000^*^	0.000	-0.6816	-0.4784
B1	B2	-0.01400	0.999	-0.1156	0.0876
	B3	-0.03400	0.934	-0.1356	0.0676
	C1	-0.08400	0.157	-0.1856	0.0176
	C2	-0.16000^*^	0.001	-0.2616	-0.0584
	C3	-0.55000^*^	0.000	-0.6516	-0.4484
B2	B3	-0.02000	0.995	-0.1216	0.0816
	C1	-0.07000	0.334	-0.1716	0.0316
	C2	-0.14600^*^	0.002	-0.2476	-0.0444
	C3	-0.53600^*^	0.000	-0.6376	-0.4344
B3	C1	-0.05000	0.706	-0.1516	0.0516
	C2	-0.12600^*^	0.008	-0.2276	-0.0244
	C3	-0.51600^*^	0.000	-0.6176	-0.4144
C1	C2	-0.07600	0.247	-0.1776	0.0256
	C3	-0.46600^*^	0.000	-0.5676	-0.3644
C2	C3	-0.39000^*^	0.000	-0.4916	-0.2884

## Discussion

The continuous advancements in acrylic denture base materials focus on two main objectives, i.e., enhancing fungal characteristics and improving mechanical properties. In this research, DMAHDM was added alone and in combination with nystatin at different concentrations to PMMA resin. DMAHDM and nystatin have been proven to enhance the antifungal properties of PMMA [1]. Material hardness is a fundamental property reflecting its resistance to deformation, which is particularly relevant in describing wear resistance [17]. In the context of acrylic resin dentures, deterioration in surface hardness over time leads to heightened mechanical and chemical wear of denture components during function and cleaning, posing significant challenges [11].

Hardness testing, particularly through the Vickers microhardness test, has long been utilized to predict wear in dental materials. This method proves reliable in evaluating stiff polymers by measuring their resistance to specific load penetration. It offers a convenient and efficient way to assess the conversion degree in such materials [18].

This study shows that incorporating DMAHDM into PMMA increases surface hardness compared to the unmodified control; however, concentrations ≥10% diminish the hardness gain relative to 5% DMAHDM. It is anticipated that the incomplete polymerization of PMMA denture base material after adding DMAHDM may result in residual monomer, adversely affecting mechanical properties due to a plasticizing effect [19]. Additionally, DMAHDM concentrations beyond saturation act as impurities within the resin matrix, weakening the material and impairing its mechanical properties. This saturation limit could explain observed reductions in hardness [20]. Variations in outcomes across studies may stem from differences in antimicrobial agents, concentrations, addition methods, or PMMA denture base materials used. Adding nystatin tended to reduce hardness modestly vs. DMAHDM alone at the same concentration. The antimicrobial agent was added physically, and there is expected to be no chemical linkage with the polymeric matrix, therefore leading to potential leakage. The decrease in surface hardness may be attributed to insufficient bonding between PMMA resin and nystatin [21].

Understanding water sorption in modified PMMA resin with DMAHDM and DMAHDM plus nystatin is crucial for clinical applications, as water uptake can impact dimensional stability and other physical properties of denture bases. According to ISO 1567, water sorption values for denture base materials should not exceed 32 µg/mm³ (22.8 µg/mm²) after one week of storage, a criterion all specimens in both control and experimental groups met. Previous research has indicated that the water sorption of different resin types typically falls within the range of 10-32 g/mm³. The current study's findings align with this range, confirming the consistency of the PMMA resin used. The control group exhibited notably lower water sorption than the experimental groups [22].

Compared to unaged and 30-day-aged specimens without antifungal agents, all heat-cured PMMA resin containing DMAHDM alone or combined with nystatin exhibited higher water sorption. The increases in water sorption and solubility with higher DMAHDM loadings likely relate to the hydrophilic QAM functionality and potential alterations in network architecture. Makvandi et al. similarly highlighted that water sorption increased with the addition of DMAHDM in antifungal agent-reinforced polymer [23].

Water absorption in PMMA resin is influenced by the polarity of PMMA molecules and the diffusion of water molecules between polymer chains. Adding an antifungal agent increases resin polarity, leading to higher water sorption, affecting mechanical properties, and causing resin expansion. While controlled water absorption can compensate for polymerization shrinkage, excessive absorption can be detrimental, potentially causing long-term plasticization effects and relaxation of internal polymerization stresses over time [24]. Increasing the DMAHDM concentration from 5% to 20% in unaged and aged specimens led to higher solubility in water. Among the experimental groups, specimens with 20% DMAHDM combined with nystatin exhibited the highest water solubility, followed by those with 10% DMAHDM combined with nystatin. This increased solubility can be attributed to the presence of nystatin between the polymer chains of the PMMA resin, which separates the chains and increases the space between them [25].

Solubility in water may stem from unreacted compounds and the solubility of impregnated antifungal agents. As nystatin particles leach out of the polymer, they may create voids that absorb more water, increasing water uptake and enhancing nystatin release [26]. Consequently, materials containing nystatin and DMAHDM are useful for treating denture-related stomatitis-adding DMAHDM alone or combined with nystatin up to 20 wt.% resulted in minimal solubility of the PMMA resin. Additionally, all specimens in both control and experimental groups met ISO 1567 standards for water solubility. Consequently, it is concluded that DMAHDM alone or in combination with nystatin has been effectively integrated into the network structure of the PMMA resin.

Adding small quantities of antimicrobial agents to PMMA resin may offer benefits in microbial control, with minimal impact on mechanical properties compared to potential advantages, particularly for patients with inadequate denture cleaning habits. This modification could be particularly advantageous for elderly individuals with limited physical or cognitive abilities.

The sample size is small; hence, a larger sample size would lead to more reliable conclusions. The study duration is limited to 1-30 days, which would not be conclusive to provide complete insight into material degradation, antimicrobial efficacy over time, and potential leaching of DMAHDM and nystatin. This in vitro study may not fully capture the complexities of the oral environment, including factors like temperature changes, salivary enzymes, and microbial biofilm formation. In vivo studies are necessary to confirm clinical relevance. While hardness was evaluated, other important mechanical properties, such as flexural strength, impact resistance, and fatigue behavior, were not assessed. These factors are crucial for determining the durability of denture materials. Although the study includes antimicrobial agents (DMAHDM and nystatin), it does not directly evaluate their effectiveness against common oral pathogens, leaving a gap in understanding their clinical benefits. The study does not address the potential cytotoxic effects of DMAHDM and nystatin on oral tissues. Future research should include biocompatibility testing to ensure patient safety. Changes in color, translucency, or handling characteristics of the resin due to the incorporation of DMAHDM and nystatin were not considered, which could affect patient satisfaction and the ease of use for dental professionals.

## Conclusions

All DMAHDM‑modified formulations increased hardness versus control, with 5% DMAHDM achieving the highest values. Increasing DMAHDM concentration and adding nystatin raised water sorption and solubility, negatively affecting long‑term stability. These findings highlight the importance of optimizing additive concentrations to balance mechanical performance and material durability in resin-based dental applications. Future work should prioritize concentration optimization and undertake comprehensive evaluations under clinically relevant simulated oral environments to validate the long-term durability and functional performance of the modified materials.
